# Summary of Guidance for Public Health Strategies to Address High Levels of Community Transmission of SARS-CoV-2 and Related Deaths, December 2020

**DOI:** 10.15585/mmwr.mm6949e2

**Published:** 2020-12-11

**Authors:** Margaret A. Honein, Athalia Christie, Dale A. Rose, John T. Brooks, Dana Meaney-Delman, Amanda Cohn, Erin K. Sauber-Schatz, Allison Walker, L. Clifford McDonald, Leandris C. Liburd, Jeffrey E. Hall, Alicia M. Fry, Aron J. Hall, Neil Gupta, Wendi L. Kuhnert, Paula W. Yoon, Adi V. Gundlapalli, Michael J. Beach, Henry T. Walke, CDC COVID-19 Response Team, CDC COVID-19 Response Team, Eduardo Azziz-Baumgartner, Sarah Bennett, Chris Braden, Jennifer Buigut, Tom Chiller, Cindy R. Friedman, Carolyn M. Greene, Olga Henao, Christine Kosmos, Adam MacNeil, Barbara Marston, Greta Massetti, Jose Montero, Cria G. Perrine, Kara Polen, Karen Remley, Reynolds Salerno, Kelly A. Shaw, Ian Williams

**Affiliations:** 1 CDC COVID-19 Emergency Response.; CDC; CDC; CDC; CDC; CDC; CDC; CDC; CDC; CDC; CDC; CDC; CDC; CDC; CDC; CDC; CDC; CDC; CDC; CDC.

In the 10 months since the first confirmed case of coronavirus disease 2019 (COVID-19) was reported in the United States on January 20, 2020 (*1*), approximately 13.8 million cases and 272,525 deaths have been reported in the United States. On October 30, the number of new cases reported in the United States in a single day exceeded 100,000 for the first time, and by December 2 had reached a daily high of 196,227.* With colder weather, more time spent indoors, the ongoing U.S. holiday season, and silent spread of disease, with approximately 50% of transmission from asymptomatic persons (*2*), the United States has entered a phase of high-level transmission where a multipronged approach to implementing all evidence-based public health strategies at both the individual and community levels is essential. This summary guidance highlights critical evidence-based CDC recommendations and sustainable strategies to reduce COVID-19 transmission. These strategies include 1) universal face mask use, 2) maintaining physical distance from other persons and limiting in-person contacts, 3) avoiding nonessential indoor spaces and crowded outdoor spaces, 4) increasing testing to rapidly identify and isolate infected persons, 5) promptly identifying, quarantining, and testing close contacts of persons with known COVID-19, 6) safeguarding persons most at risk for severe illness or death from infection with SARS-CoV-2, the virus that causes COVID-19, 7) protecting essential workers with provision of adequate personal protective equipment and safe work practices, 8) postponing travel, 9) increasing room air ventilation and enhancing hand hygiene and environmental disinfection, and 10) achieving widespread availability and high community coverage with effective COVID-19 vaccines. In combination, these strategies can reduce SARS-CoV-2 transmission, long-term sequelae or disability, and death, and mitigate the pandemic’s economic impact. Consistent implementation of these strategies improves health equity, preserves health care capacity, maintains the function of essential businesses, and supports the availability of in-person instruction for kindergarten through grade 12 schools and preschool. Individual persons, households, and communities should take these actions now to reduce SARS-CoV-2 transmission from its current high level. These actions will provide a bridge to a future with wide availability and high community coverage of effective vaccines, when safe return to more everyday activities in a range of settings will be possible.

## Recommended Public Health Strategies

**Universal use of face masks.** Consistent and correct use of face masks is a public health strategy critical to reducing respiratory transmission of SARS-CoV-2, particularly in light of estimates that approximately one half of new infections are transmitted by persons who have no symptoms (*2*,*3*). Compelling evidence now supports the benefits of cloth face masks for both source control (to protect others) and, to a lesser extent, protection of the wearer.^†^ To preserve the supply of N95 respirators for health care workers and other medical first responders, CDC recommends nonvalved, multilayer cloth masks or nonmedical disposable masks for community use.^§^ Face mask use is most important in indoor spaces and outdoors when physical distance of ≥6 feet cannot be maintained. Within households, face masks should be used when a member of the household is infected or has had recent potential COVID-19 exposure (e.g., known close contact or potential exposure related to occupation, crowded public settings, travel, or nonhousehold members in your house). A community-level plan for distribution of face masks to specific populations, such as those who might experience barriers to access, should be developed (Table).

**TABLE:** Individual- and community-level public health strategies to reduce SARS-CoV-2 transmission*

Recommended public health strategies	Individual- and household-level strategies	Community-level strategies (at state or local level)	Links to guidance
Universal use of face masks	Consistent and correct use of face masks, including within the household if there is a COVID-19 case or a person with a known or possible exposure in the household	Issue policies or directives mandating universal use of face masks in indoor (nonhousehold) settings	Considerations for wearing masks: https://www.cdc.gov/coronavirus/2019-ncov/prevent-getting-sick/cloth-face-cover-guidance.html
		Plan for provision of face masks for specific populations if needed	Caring for someone sick at home, when to wear a mask or gloves: https://www.cdc.gov/coronavirus/2019-ncov/if-you-are-sick/care-for-someone.html#face-covering
			Protect your home: https://www.cdc.gov/coronavirus/2019-ncov/prevent-getting-sick/protect-your-home.html
Physical distancing and limiting contacts	Maintain physical distance (≥6 feet) from other persons when possible, and limit number of contacts with persons outside the immediate household	Physical barriers and visual reminders might promote adherence to maintaining physical distance	Social distancing: https://www.cdc.gov/coronavirus/2019-ncov/prevent-getting-sick/social-distancing.html
			Personal and social activities: https://www.cdc.gov/coronavirus/2019-ncov/daily-life-coping/personal-social-activities.html
Avoid nonessential indoor spaces and crowded outdoor settings	Avoid nonessential indoor spaces and crowded outdoor settings	Issue policies or directives restricting some nonessential indoor spaces that pose the highest risk for transmission	Daily activities and going out: https://www.cdc.gov/coronavirus/2019-ncov/daily-life-coping/going-out.html
		Promoting flexible worksites (e.g., telework); apply limits to occupancy of indoor spaces and to the size of social gatherings	Considerations for events and gatherings: https://www.cdc.gov/coronavirus/2019-ncov/community/large-events/considerations-for-events-gatherings.html
Increased testing, diagnosis, and isolation	Persons with a known exposure to someone with COVID-19, with possible exposure, or who experience symptoms should promptly seek testing; symptomatic or infected persons should isolate promptly; exposed persons should quarantine	Increase access to testing, including expanded screening testing of prioritized persons/groups, prioritizing those with many interactions (or interactions with persons at high risk) based on their occupational or residential setting	Testing: https://www.cdc.gov/coronavirus/2019-ncov/testing/index.html
			Expanded screening testing: https://www.cdc.gov/coronavirus/2019-ncov/php/open-america/expanded-screening-testing.html
		Promptly report test results to the person tested and to public health authorities	Isolate if you are sick: https://www.cdc.gov/coronavirus/2019-ncov/if-you-are-sick/isolation.html
			Guidance for health departments about COVID-19 testing in the community: https://www.cdc.gov/coronavirus/2019-ncov/php/open-america/testing.html
Prompt case investigation and contact tracing to identify, quarantine, and test close contacts	Persons with diagnosed COVID-19 should provide names of known contacts; close contacts should anticipate a call from the health department, answer the call, adhere to quarantine, seek testing, and encourage their household members to quarantine	When incidence is high and overwhelms capacity, prioritize case investigation and contact tracing to promptly quarantine and test close contacts, based on time since sample collection and risk for spread to others (e.g., those working in high-density settings)	When to quarantine: https://www.cdc.gov/coronavirus/2019-ncov/if-you-are-sick/quarantine.html
			Contact tracing (your health): https://www.cdc.gov/coronavirus/2019-ncov/daily-life-coping/contact-tracing.html
			Contact tracing (health departments): https://www.cdc.gov/coronavirus/2019-ncov/php/open-america/contact-tracing/index.html
			Prioritizing case investigation and contact tracing: https://www.cdc.gov/coronavirus/2019-ncov/php/contact-tracing/contact-tracing-plan/prioritization.html
			Quarantine: https://www.cdc.gov/coronavirus/2019-ncov/more/scientific-brief-options-to-reduce-quarantine.html
Safeguarding persons most at risk for severe illness or death	Persons with underlying medical conditions or risk factors that place them at increased risk for severe illness or death should minimize contact with nonhousehold members and nonessential indoor spaces	Protect persons most at risk for severe illness or death through 1) identifying populations at high risk in the community and 2) expanding access to testing, provision of support services, and messaging	People at increased risk: https://www.cdc.gov/coronavirus/2019-ncov/need-extra-precautions/index.html
Protecting essential workers	Essential workers should employ all available public health strategies to reduce their risk (e.g., wear face masks and keep physical distance)	Protect essential workers through policies directing administrative and structural prevention as well as expanded testing	Essential services and critical infrastructure: https://www.cdc.gov/coronavirus/2019-ncov/community/workplaces-businesses/essential-services.html
			COVID-19 critical infrastructure sector response planning: https://www.cdc.gov/coronavirus/2019-ncov/community/critical-infrastructure-sectors.html
			CISA guidance on the essential critical infrastructure workforce: https://www.cisa.gov/publication/guidance-essential-critical-infrastructure-workforce
Postponing travel	Travel should be postponed. Those who choose to travel internationally should be tested with a viral test 1–3 days before departure and retested 3–5 days after arrival; domestic travelers should also consider getting tested	Issue policies or directives mandating universal use of face masks on all modes of public transportation	Travel: https://www.cdc.gov/coronavirus/2019-ncov/travelers/index.html
	Travelers should stay home or reduce nonessential activities before and after travel and be diligent about mask wearing, physical distancing, hand hygiene, and symptom monitoring		When not to travel: https://www.cdc.gov/coronavirus/2019-ncov/travelers/when-to-delay-travel.html
			Wear face masks on public transportation conveyances and at transportation hubs: https://www.cdc.gov/coronavirus/2019-ncov/travelers/face-masks-public-transportation.html
			Mask and travel guidance: https://www.cdc.gov/quarantine/masks/mask-travel-guidance.html
			Domestic travel: https://www.cdc.gov/coronavirus/2019-ncov/travelers/travel-during-covid19.html
			Testing and international air travel: https://www.cdc.gov/coronavirus/2019-ncov/travelers/testing-air-travel.html
Increased room air ventilation, enhanced hand hygiene, and cleaning and disinfection	Increase room air ventilation	Enhance ventilation and cleaning and disinfection, particularly of essential indoor spaces	SARS-CoV-2 and potential airborne transmission: https://www.cdc.gov/coronavirus/2019-ncov/more/scientific-brief-sars-cov-2.html
	Frequent handwashing	Ensure provision of adequate hand sanitation supplies	Ventilation: https://www.cdc.gov/coronavirus/2019-ncov/community/general-business-faq.html#Ventilation
			When and how to wash your hands: https://www.cdc.gov/handwashing/when-how-handwashing.html
			Cleaning and disinfecting: https://www.cdc.gov/coronavirus/2019-ncov/community/clean-disinfect/index.html
Widespread availability and coverage with effective vaccines	Seek vaccine when appropriate following ACIP recommendations	Plan for distribution and administration of vaccines to achieve high community coverage	Vaccines: https://www.cdc.gov/coronavirus/2019-ncov/vaccines/index.html
	Continue to follow all mitigation measures until community vaccination coverage is adequate	Communicate that mitigation measures still need to be followed until community vaccination coverage is determined to be adequate	Vaccination planning: https://www.cdc.gov/vaccines/covid-19/planning/index.html

**Abbreviations:** ACIP = Advisory Committee on Immunization Practices; COVID-19 = coronavirus disease 2019.

* https://www.cdc.gov/coronavirus/2019-ncov/communication/guidance-list.html.

**Physical distancing and limiting contacts.** Maintaining physical distance (≥6 feet) lowers the risk for SARS-CoV-2 infection through exposure to infectious respiratory droplets and aerosols and is important, even if no symptoms are apparent, because transmission can occur from asymptomatic infected persons^¶^ (*2*,*3*). Outside the household setting, close physical contact, shared meals, and being in enclosed spaces have all been associated with an increased infection risk (*4*–*7*). Although the impact of physical distancing is difficult to disaggregate from other interventions, one study estimated that physical distancing decreased the average number of daily contacts by as much as 74% and reduced the reproductive number (R_0_, a measure of transmission, which describes the average number of persons infected by one infectious person) to <1 (*8*). Because the highest risk for transmission has been documented among household contacts of COVID-19 patients (*9*), keeping the household safe requires physical distancing, using the other public health strategies summarized here, and, in particular, consistent and correct use of face masks (outside the household and in some circumstances within the household) to prevent introduction and transmission of SARS-CoV-2. At the community level, physical barriers and visual reminders might promote adherence to maintaining physical distance.

**Avoiding nonessential indoor spaces and crowded outdoor settings.** Exposures at nonessential indoor settings and crowded outdoor settings pose a preventable risk to all participants.**^,^^††^ Indoor venues, where distancing is not maintained and consistent use of face masks is not possible (e.g., restaurant dining), have been identified as particularly high-risk scenarios (*7*,*10*). Crowded events in outdoor settings have also been linked to spread of SARS-CoV-2, although it can be difficult to isolate the impact of crowded outdoor events from related indoor social interactions (*11*). To reduce risk, some restaurants are providing take-away service and well-ventilated open-air dining, and in many cases, exercise or physical activity (individual or group) can be moved to outdoor settings where physical distance is maintained and face masks are worn. Community-level policies can further reduce transmission by promoting flexible worksites (e.g., telework) and hours, as well as by applying limits to occupancy of indoor spaces and to the size of social gatherings.

**Increased testing, diagnosis, and isolation.** Isolation is used to separate persons infected with SARS-CoV-2 from those who are not infected; persons who are identified by testing to be infected should be rapidly isolated.^§§^ Estimates vary, however, >40% of persons infected with SARS-CoV-2 might be asymptomatic, and transmission from presymptomatic persons (those who are not symptomatic at the time they transmit infection, but who later experience symptoms) and asymptomatic persons (infected persons who never experience symptoms) is estimated to account for >50% of all transmission (*2**,**3*). Therefore, reliance on symptom screening to identify infected persons is inadequate (*12*). Increased testing is an important strategy to interrupt silent transmission of SARS-CoV-2 from asymptomatic and presymptomatic persons. However, because the sensitivity of available tests and the time since exposure varies, a negative test might provide false reassurance; thus, all prevention strategies should continue to be followed including use of face masks and maintaining physical distance. A comparative analysis of data from six large countries demonstrated that high levels of testing, combined with robust contact tracing, can substantially reduce the transmission of SARS-CoV-2 (*13*). Frequent testing and contact tracing, combined with other mitigation measures, effectively limited SARS-CoV-2 transmission on a college campus (*14*). In addition to testing symptomatic persons and those with known exposure, a strategy of routinely testing certain population groups with high numbers of interactions with other persons, based on their occupational or residential setting, can more rapidly identify asymptomatic and presymptomatic infectious persons and their close contacts for isolation and quarantine.^¶¶^ Communities with high or increasing SARS-CoV-2 transmission should increase screening testing, focusing on persons at increased risk for exposure (e.g., workers in high-density worksites) or persons who might have the potential to transmit infection to large numbers of other persons (e.g., persons working in congregate settings) or to transmit to persons at risk for severe COVID-19–associated illness or death (e.g., staff members in nursing homes). Expanded screening testing should be implemented in a manner that promotes health equity for persons with limited resources or other barriers to accessing health care. In addition, prompt reporting of test results to the person tested and to public health authorities can facilitate rapid isolation, case investigation and contact tracing, and accurate monitoring of COVID-19 in the community.

**Prompt case investigation and contact tracing to identify, quarantine, and test close contacts.** Case investigation is the process of obtaining comprehensive information about persons with a diagnosis of COVID-19 and is followed by contact tracing, which includes identifying and communicating with persons exposed to SARS-CoV-2 (i.e., close contacts***) to inform them of their exposure, educate them about risks for and symptoms of COVID-19, and encourage them to quarantine, seek testing, and monitor themselves for signs or symptoms of illness.^†††^ Quarantine is used to keep a person who was exposed to SARS-CoV-2 away from others.^§§§^ Contact tracing is most feasible when the incidence of COVID-19 in the community or workplace is low or declining, when testing and reporting of results can occur quickly (*15*), and when most contacts can be reached and quarantined (*16*). When one or more of these conditions is not met or when local capacity is overwhelmed, health departments should narrow the scope of contact tracing activities and emphasize community mitigation measures. Investigations should prioritize persons who most recently received positive SARS-CoV-2 test results, as well as identify and quarantine household contacts and persons exposed in a congregate living facility, high-density workplace, or other setting (or event) with potential extensive transmission.^¶¶¶^ Because the risk for household transmission is high and occurs rapidly in the absence of face masks or other protective behaviors, household members of persons with diagnosed COVID-19 should be quarantined, and, in the event that they experience symptoms or receive a positive test result, they should be isolated (*9*,*17*). Eliciting and reaching contacts in a timely manner is challenging (*18*,*19*), and quarantine can impose economic and financial burdens (*20*); adherence to quarantine might require provision of appropriate support services.**** Persons who receive positive SARS-COV-2 test results should also be encouraged to serve as their own contact tracers by informing close contacts that they have been exposed and encouraging those persons to quarantine, monitor for symptoms, and seek testing.

**Safeguarding persons most at risk for severe illness or death**. To protect those who are at highest risk for severe COVID-19–associated outcomes, universal mitigation efforts are needed. SARS-CoV-2 infection can be completely asymptomatic or can manifest as a life-threatening illness; disease can result in postacute and long-term sequelae or disability among survivors. Risk for severe illness increases with age and is highest for those aged ≥85 years.^††††^ In the United States, approximately 80% of reported COVID-19 deaths have occurred in patients aged ≥65 years.

Certain underlying medical conditions also increase risk for severe illness or death for persons of any age with COVID-19.^§§§§^ Long-term care facilities serve older adults and persons with complex medical conditions; COVID-19 can spread rapidly in these congregate settings, resulting in high rates of morbidity and mortality. To prevent introduction and transmission of SARS-CoV-2, these facilities should implement strict infection prevention and control measures and expanded screening testing of both staff members and residents to rapidly identify and isolate infected persons.^¶¶¶¶^

COVID-19 has also disproportionately affected racial and ethnic minority groups.***** An age-standardized analysis of COVID-19–associated deaths reported to the National Vital Statistics System through November 25, 2020, found that Black persons accounted for 26.9% of COVID-19–related deaths, despite representing 12.7% of the U.S. population.^†††††^ Persons who belong to racial or ethnic minority groups are likewise disproportionately affected by the underlying medical conditions that increase risk for severe COVID-19 illness and death, likely because of long-standing inequities in social determinants of health. Members of racial or ethnic minority groups are more likely to experience lower socioeconomic status, to live in crowded housing, and possibly to be employed in occupations that require in-person work.^§§§§§^ In addition, access to health care might be limited, including obtaining testing and care for COVID-19.

Persons who are at highest risk for severe COVID-19–associated illness or death or who share a household with someone at high risk should minimize their individual and household risk by avoiding nonessential interactions with persons outside their household whenever possible and implementing all recommended public health prevention strategies. Some approaches to safeguarding those with underlying medical conditions include promoting access to and use of telehealth when feasible and appropriate, use of no-contact pickup for groceries or other essential items, and use of online (versus in-person) educational instruction.

**Protecting essential workers**. Essential (critical infrastructure) workers include health care personnel and employees in other essential workplaces (e.g., first responders and grocery store workers).^¶¶¶¶¶^ Protecting essential workers requires full implementation of all evidence-based strategies outlined in this guidance. When a COVID-19 vaccine is authorized for use by the Food and Drug Administration (FDA) and recommended by the Advisory Committee on Immunization Practices (ACIP), essential workers, including health care personnel, are among the populations being considered for initial phased allocation of limited vaccine doses (*21*). Implementation of infection prevention and control with adequate supplies and extensive use of telehealth options and nurse-directed triage of patients, as well as screening of all persons entering health care facilities for signs and symptoms of COVID-19, can protect health care personnel and reduce risk for SARS-CoV-2 transmission in health care facilities.****** U.S. food manufacturing and agriculture is another sector that has been substantially affected by COVID-19, especially among workers in meat and poultry processing facilities, with disproportionate effects among persons who belong to racial or ethnic minority groups (*22*). CDC and the Occupational Safety and Health Administration released guidance on administrative and engineering controls that should be part of COVID-19 assessment and control plans for these workplaces.^††††††^ When cessation of operation of a facility might cause serious harm or danger to public health or safety, essential workers who are known close contacts of persons with confirmed COVID-19 might need to return to work as a last resort; however, if they return to work, they should use face masks and maintain physical distancing, and the workplace should be appropriately disinfected.^§§§§§§^ These persons should only return to work if they are and remain asymptomatic and undergo at least daily active symptom monitoring with immediate removal from the workplace if any signs or symptoms of possible COVID-19 occur; viral testing of all close contacts is recommended, and those with positive test results should not return to work.

**Postponing travel**. Travel increases the likelihood of SARS-CoV-2 exposure and infection and could translocate infection between communities. Postponing travel is the best way to reduce this risk.^¶¶¶¶¶¶^ Any traveler who is symptomatic, has had close contact with a person with COVID-19 and has not met criteria for release from quarantine, or has a positive or pending SARS-CoV-2 test result should not travel.******* For those contemplating international travel, CDC recommends getting tested with a viral test for SARS-CoV-2 1–3 days before departure and getting retested 3–5 days after arrival.^†††††††^ Domestic travelers should also consider testing. Testing does not eliminate all risk and should be combined with other recommended public health strategies. Both domestic and international travelers should stay home or reduce nonessential activities before travel, and for 7 days after travel if tested, even if test results are negative. If not tested, this period should be extended to 10 days. Travelers should be diligent about mask wearing, physical distancing, hand hygiene, and symptom monitoring. For 14 days after arrival, travelers should avoid close contact with persons at higher risk for severe COVID-19–associated outcomes and wear masks in household spaces shared with those who did not travel.

**Increased room air ventilation, enhanced hand hygiene, and cleaning and disinfection**. Increasing room air ventilation, enhancing hand hygiene, and cleaning and disinfecting frequently touched surfaces might help decrease transmission of SARS-CoV-2 (*23*).^§§§§§§§^ Although the epidemiology of SARS-CoV-2 suggests that most transmission is close person-to-person, there have been some documented cases of presumed airborne transmission.^¶¶¶¶¶¶¶^ Avoiding nonessential indoor spaces can help reduce this risk. For indoor settings, increased room air ventilation can decrease the concentration of small droplets and particles carrying infectious virus suspended in the air and, thereby, presumably decrease the risk for transmission.******** Hand hygiene includes handwashing with soap and water or using alcohol-based hand sanitizer.^††††††††^ Handwashing mechanically removes pathogens, and laboratory data demonstrate that hand sanitizers that contain at least 60% alcohol inactivate SARS-CoV-2 (*24*). These strategies, combined with appropriate cleaning and disinfection of surfaces, might prevent indirect transmission through touching surfaces contaminated with virus from an infected person, followed by touching the mouth, nose, or eyes.

**Widespread availability and use of effective vaccines**. Widespread availability and high community coverage with safe and effective COVID-19 vaccines represent the most important public health strategy to control the pandemic. Many COVID-19 vaccine candidates are currently in clinical trials. Promising products are being manufactured in anticipation of Emergency Use Authorization from the FDA. The federal government has established a centralized system to order, distribute, and track COVID-19 vaccines through states, tribal nations, and territories; these jurisdictions are preparing for vaccination with extensive planning for vaccine distribution and administration.^§§§§§§§§^ After FDA authorization of the use of one or more COVID-19 vaccines in the United States, the ACIP will review safety and efficacy data for each of the authorized vaccines and will issue recommendations for use to ensure equitable access (*21**,**25*). Ensuring transparency in these efforts, monitoring for adverse events, and working with communities to address concerns will be critical to obtaining the confidence and trust of the public and health care providers. CDC and FDA will monitor the effectiveness and safety of all COVID-19 vaccines and update and communicate this information regularly. Vaccinated persons should continue to adhere to all mitigation measures (e.g., mask use, physical distancing, and hand hygiene) until both doses in the series have been received and the duration of immunity provided by vaccines has been sufficiently established.

## Discussion

No single strategy can control the pandemic; rather, a multipronged approach using all available evidence-based strategies at the individual and community levels can break transmission chains and address high levels of community transmission; reduce related illnesses, long-term sequelae, and deaths; and mitigate the pandemic’s economic impact. Because COVID-19 has disproportionately affected persons with certain risk factors (e.g., age and some underlying medical conditions) and racial/ethnic minorities, implementing public health prevention strategies in a manner that assures health equity is imperative to safeguard those who have borne the worst of the pandemic’s impact. The U.S. health care system is being stressed by COVID-19, with multiple jurisdictions establishing expanded or alternative treatment settings. Continuing mitigation efforts will be essential to preserve capacity for adequate treatment of persons with COVID-19 and other urgent health conditions, and to protect essential and preventive services that are not amenable to telehealth. Schools provide numerous benefits beyond education, including school meal programs and social, physical, behavioral, and mental health services. Because of their critical role for all children and the disproportionate impact that school closures can have on those with the least economic means, kindergarten through grade 12 schools should be the last settings to close after all other mitigation measures have been employed and the first to reopen when they can do so safely.^¶¶¶¶¶¶¶¶^ Similarly, full implementation of public health prevention strategies can help preserve the functioning of essential businesses that supply food to the population, contribute to the health protection of communities and individual persons, and fuel economic recovery. Full implementation of and adherence to these strategies will save lives. As communities respond to high levels of SARS-CoV-2 transmission, these strategies will also provide the necessary bridge to a future with wide availability and high levels of coverage with effective vaccines, and thereby a safe return to more everyday activities in a range of settings.

**Summary**

**What is already known about this topic?**

The United States is experiencing high levels of SARS-CoV-2 transmission.

**What is added by this report?**

COVID-19 pandemic control requires a multipronged application of evidence-based strategies while improving health equity: universal face mask use, physical distancing, avoiding nonessential indoor spaces, increasing testing, prompt quarantine of exposed persons, safeguarding those at increased risk for severe illness or death, protecting essential workers, postponing travel, enhancing ventilation and hand hygiene, and achieving widespread COVID-19 vaccination coverage.

**What are the implications for public health practice?**

These combined strategies will protect health care, essential businesses, and schools, bridging to a future with high community coverage of effective vaccines and safe return to more activities in a range of settings.

## Footnotes

* https://covid.cdc.gov/covid-data-tracker/#trends_dailytrendscases.

† https://www.cdc.gov/coronavirus/2019-ncov/more/masking-science-sars-cov2.html.

§ https://www.cdc.gov/coronavirus/2019-ncov/prevent-getting-sick/cloth-face-cover-guidance.html.

¶ https://www.cdc.gov/coronavirus/2019-ncov/prevent-getting-sick/social-distancing.html.

** https://www.cdc.gov/coronavirus/2019-ncov/community/large-events/considerations-for-events-gatherings.html.

†† https://www.cdc.gov/coronavirus/2019-ncov/daily-life-coping/going-out.html.

§§ https://www.cdc.gov/coronavirus/2019-ncov/if-you-are-sick/isolation.html.

¶¶ https://www.cdc.gov/coronavirus/2019-ncov/php/open-america/expanded-screening-testing.html.

*** https://www.cdc.gov/coronavirus/2019-ncov/php/contact-tracing/contact-tracing-plan/appendix.html#contact.

††† https://www.cdc.gov/coronavirus/2019-ncov/daily-life-coping/contact-tracing.html.

§§§ https://www.cdc.gov/coronavirus/2019-ncov/if-you-are-sick/quarantine.html.

¶¶¶ https://www.cdc.gov/coronavirus/2019-ncov/php/contact-tracing/contact-tracing-plan/prioritization.html.

**** https://www.cdc.gov/coronavirus/2019-ncov/php/contact-tracing/contact-tracing-plan/support-services.html.

†††† https://www.cdc.gov/coronavirus/2019-ncov/need-extra-precautions/older-adults.html.

§§§§ https://www.cdc.gov/coronavirus/2019-ncov/need-extra-precautions/people-with-medical-conditions.html.

¶¶¶¶ https://www.cdc.gov/coronavirus/2019-ncov/hcp/nursing-home-long-term-care.html.

***** https://www.cdc.gov/coronavirus/2019-ncov/community/health-equity/race-ethnicity.html.

††††† https://www.cdc.gov/nchs/nvss/vsrr/covid19/health_disparities.htm.

§§§§§ https://www.census.gov/content/dam/Census/programs-surveys/ahs/publications/Measuring_Overcrowding_in_Hsg.pdf.

¶¶¶¶¶ https://www.cisa.gov/publication/guidance-essential-critical-infrastructure-workforce.

****** https://www.cdc.gov/coronavirus/2019-ncov/hcp/infection-control-recommendations.html.

†††††† https://www.cdc.gov/coronavirus/2019-ncov/community/organizations/meat-poultry-processing-workers-employers.html.

§§§§§§ https://www.cdc.gov/coronavirus/2019-ncov/community/critical-infrastructure-sectors.html?CDC_AA_refVal.

¶¶¶¶¶¶
https://www.cdc.gov/coronavirus/2019-ncov/travelers/travel-during-covid19.html .

******* https://www.cdc.gov/coronavirus/2019-ncov/travelers/when-to-delay-travel.html.

††††††† https://www.cdc.gov/coronavirus/2019-ncov/travelers/testing-air-travel.html.

§§§§§§§ https://www.cdc.gov/coronavirus/2019-ncov/community/clean-disinfect/index.html.

¶¶¶¶¶¶¶ https://www.cdc.gov/coronavirus/2019-ncov/more/scientific-brief-sars-cov-2.html.

******** https://www.cdc.gov/niosh/conferences/events/heatventair.html.

†††††††† https://www.cdc.gov/coronavirus/2019-ncov/global-covid-19/handwashing.html.

¶¶¶¶¶¶¶¶ https://www.cdc.gov/coronavirus/2019-ncov/community/schools-childcare/indicators.html.
